# Buruli Ulcer Disease in Republic of the Congo

**DOI:** 10.3201/eid2006.131498

**Published:** 2014-06

**Authors:** Estelle Marion, Damas Obvala, Jeremie Babonneau, Marie Kempf, Kingsley B. Asiedu, Laurent Marsollier

**Affiliations:** Fondation Raoul Follereau, Pobè, Bénin (E. Marion);; Centre Hospitalier Universitaire d’Angers, Angers, France (E. Marion, J. Babonneau, M. Kempf, L. Marsollier);; INSERM, Angers (E. Marion, J. Babonneau, L. Marsollier);; Ministère de la Santé, Brazzaville République du Congo (D. Obvala);; World Health Organization, Geneva, Switzerland (K.B. Asiedu)

**Keywords:** Buruli ulcer, Mycobacterium ulcerans, Republic of the Congo, Congo Brazzaville, tropical disease, skin disease, necrotic disease, bacteria

**To the Editor**: Buruli ulcer, which is caused by the *Mycobacterium ulcerans* bacterium, is a severe disabling necrotic disease of the skin, occurring mainly in swampy rural areas of western and central Africa. This tropical disease is neglected, despite being the third most common mycobacterial disease of humans, after tuberculosis and leprosy. The disease has become substantially more frequent over the past decade, particularly around the Gulf of Guinea, and has been detected or suspected in at least 31 countries (*1*). Clinical diagnosis of Buruli ulcer disease should be confirmed by PCR, as recommended by the World Health Organization (WHO); and case-patients should be treated with rifampin/streptomycin daily for 8 weeks (therapy available since 2004), combined, if necessary, with surgery.

Although confirmed cases of Buruli ulcer disease have been reported in all countries neighboring the Republic of the Congo (hereafter called Congo) (*2*–*4*), only 1 report of a confirmed case in Congo has been published (*5*) (Figure, panel A). During 2007–2012, a total of 573 clinical cases of Buruli ulcer disease were reported to WHO by the National Leprosy, Buruli Ulcer and Yaws Control Program in Congo. We report 108 cases (19% of all cases reported) that were confirmed, in accordance with WHO recommendations, by quantitative PCR, the most sensitive and specific testing method available (*6*).

**Figure:**
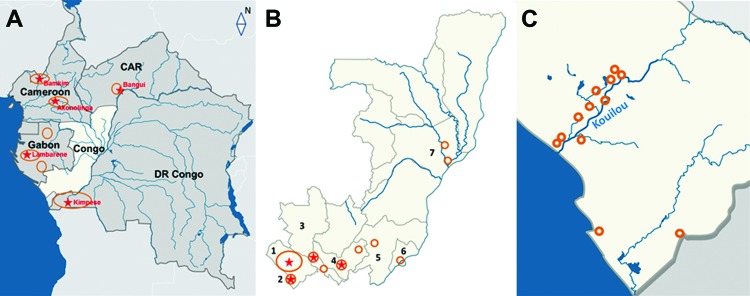
Buruli ulcer–endemic areas in the Republic of the Congo (RC) and neighboring countries. A) Buruli ulcer cases have been reported in all countries neighboring RC. CAR, Central African Republic; DR Congo, Democratic Republic of the Congo. B) RC (representing the white area in panel A). The numbers indicate the 7 departments or communes (of 12 total) where PCR-positive cases of Buruli ulcer disease were diagnosed. 1, Kouilou Department; 2 Pointe Noire Commune; 3, Niari Department; 4, Bouenza Department; 5, Pool Department; 6, Brazzaville Commune; 7, Cuvette Department. C) Kouilou Department (department 1 in panel B). Most Buruli ulcer case-patients from Kouilou Department were living close to the Kouilou River. Stars indicate locations of health centers that treat Buruli ulcer disease; circles indicate areas where persons with Buruli ulcer disease were identified.

The National Leprosy, Buruli Ulcer, and Yaws Control Program, with the support of the Raoul Follereau Foundation (Paris, France), performed passive and active surveillance of Buruli ulcer in Congo during 2007–2012. Fine-needle aspirate or swab samples were obtained from patients with suspected Buruli ulcer and sent to Angers University Hospital (Angers, France) for confirmation by quantitative PCR as described (*6*,*7*). Of the 283 samples analyzed, 114 (40%) from 108 different patients were PCR positive. Of the 114 PCR-positive samples, 20 (18%) were fine-needle aspirate samples and 94 (82%) were swab samples (at least 2 swabs/lesion). The 108 case-patients included 60 (56%) female and 48 (44%) male patients; 56% of the case-patients were <15 years of age. The most common clinical form of the disease (86% of cases) was the ulcerative stage with edema or plaque. All confirmed Buruli ulcer case-patients were treated in accordance with WHO recommendations (*8*): antibiotic treatment (rifampin/streptomycin) plus surgery if necessary. All patients with nonconfirmed cases were treated according to the alternative diagnosis reached by the clinician.

Our findings show that Buruli ulcer disease affects persons in several of Congo’s administrative divisions (Figure, panel B); of the 108 patients, 77 (71%) were from Kouilou Department (Figure, panel C). The village of residence was recorded for 55of these 77 patients, 46 (84%) of whom lived in 9 villages along the Kouilou River, encompassing an area of ≈50 km × 20 km: Madingo-Kayes, Kanga, Loukouala, Mfilou, Koubotchi, Mboukoumassi, Tchisseka, Magne, and Loaka villages. This disease-endemic area includes 2 lakes, Dinga and Nanga, both of which are fed by the Kouilou River. The remaining 31 (29%) confirmed patients (i.e., those not living in Kouilou Department) lived in Niari Department (9%), Bouenza Department (6.5%), Pool Department (3%), or Cuvette Department (5.5%) or in Pointe Noire Commune (2%) or Brazzaville Commune (3%) (Figure, panel B).

The distribution of Buruli ulcer cases in Congo is unusual. The Kouilou River region was most affected, but several other areas, all in southern Congo, have confirmed Buruli ulcer patients. Cuvette Department is the 1 exception; although it is in northeastern Congo, this department did have a cluster of cases. The cases in Cuvette were identified (and the infections were diagnosed and treated) during active research into Buruli ulcer during 2009–2010. (Note that there has been no survey in this region since 2010.)

Buruli ulcer is also endemic in some areas of the countries neighboring Congo. In the Democratic Republic of the Congo, the disease is highly endemic in the Bas Congo region, which shares a border with departments in southern Congo where the disease is endemic (*9*). By contrast, the small cluster of cases diagnosed in Cuvette Department in northeastern Congo seems to be isolated from other areas where the disease is known to be endemic.

*M. ulcerans* is known to be associated with wetlands, and the Kouilou River environment is certainly suitable for its spread (*10*). Identification of this zone as a high-risk area for Buruli ulcer disease will help the Ministry of Health improve early detection, biological confirmation, and treatment programs. In the other regions, active and continuous surveillance is necessary to establish a detailed map of the villages and areas where Buruli ulcer disease is endemic; such information would enable the implementation of targeted control activities. However, active surveillance in Congo has substantially declined since 2011. Our findings support the reactivation of such surveillance campaigns to ensure the early identification and confirmation of Buruli ulcer cases and to improve patient management.
